# Short- and Long-Term Predicted and Witnessed Consequences of Digital Surveillance During the COVID-19 Pandemic: Scoping Review

**DOI:** 10.2196/47154

**Published:** 2024-05-24

**Authors:** Leigha Comer, Lorie Donelle, Bradley Hiebert, Maxwell J Smith, Anita Kothari, Saverio Stranges, Jason Gilliland, Jed Long, Jacquelyn Burkell, Jacob J Shelley, Jodi Hall, James Shelley, Tommy Cooke, Marionette Ngole Dione, Danica Facca

**Affiliations:** 1 Arthur Labatt Family School of Nursing Western University London, ON Canada; 2 School of Nursing University of South Carolina Columbia, SC United States; 3 School of Health Studies Western University London, ON Canada; 4 Department of Epidemiology and Biostatistics Schulich School of Medicine & Dentistry Western University London, ON Canada; 5 Departments of Family Medicine and Medicine Schulich School of Medicine & Dentistry Western University London, ON Canada; 6 The Africa Institute Western University London, ON Canada; 7 Department of Clinical Medicine and Surgery University of Naples Federico II Naples Italy; 8 Department of Geography and Environment Western University London, ON Canada; 9 Faculty of Information and Media Studies Western University London, ON Canada; 10 Western Law Western University London, ON Canada; 11 Faculty of Health Sciences Western University London, ON Canada; 12 Surveillance Studies Centre Queen's University Kingston, ON Canada

**Keywords:** digital surveillance, COVID-19, public health, scoping review, pandemic, digital technologies

## Abstract

**Background:**

The COVID-19 pandemic has prompted the deployment of digital technologies for public health surveillance globally. The rapid development and use of these technologies have curtailed opportunities to fully consider their potential impacts (eg, for human rights, civil liberties, privacy, and marginalization of vulnerable groups).

**Objective:**

We conducted a scoping review of peer-reviewed and gray literature to identify the types and applications of digital technologies used for surveillance during the COVID-19 pandemic and the predicted and witnessed consequences of digital surveillance.

**Methods:**

Our methodology was informed by the 5-stage methodological framework to guide scoping reviews: identifying the research question; identifying relevant studies; study selection; charting the data; and collating, summarizing, and reporting the findings. We conducted a search of peer-reviewed and gray literature published between December 1, 2019, and December 31, 2020. We focused on the first year of the pandemic to provide a snapshot of the questions, concerns, findings, and discussions emerging from peer-reviewed and gray literature during this pivotal first year of the pandemic. Our review followed the PRISMA-ScR (Preferred Reporting Items for Systematic Reviews and Meta-Analyses Extension for Scoping Reviews) reporting guidelines.

**Results:**

We reviewed a total of 147 peer-reviewed and 79 gray literature publications. Based on our analysis of these publications, we identified a total of 90 countries and regions where digital technologies were used for public health surveillance during the COVID-19 pandemic. Some of the most frequently used technologies included mobile phone apps, location-tracking technologies, drones, temperature-scanning technologies, and wearable devices. We also found that the literature raised concerns regarding the implications of digital surveillance in relation to data security and privacy, function creep and mission creep, private sector involvement in surveillance, human rights, civil liberties, and impacts on marginalized groups. Finally, we identified recommendations for ethical digital technology design and use, including proportionality, transparency, purpose limitation, protecting privacy and security, and accountability.

**Conclusions:**

A wide range of digital technologies was used worldwide to support public health surveillance during the COVID-19 pandemic. The findings of our analysis highlight the importance of considering short- and long-term consequences of digital surveillance not only during the COVID-19 pandemic but also for future public health crises. These findings also demonstrate the ways in which digital surveillance has rendered visible the shifting and blurred boundaries between public health surveillance and other forms of surveillance, particularly given the ubiquitous nature of digital surveillance.

**International Registered Report Identifier (IRRID):**

RR2-https://doi.org/10.1136/bmjopen-2021-053962

## Introduction

Public health surveillance has been described as one of the most critical functions and mechanisms of public health [1]. With a history dating back to Snow’s pioneering investigations into cholera epidemics in England in the 19th century, surveillance has long been leveraged as a tool for meeting the objectives of public health, including population health assessment, health surveillance, health promotion, disease and injury prevention, and health protection [1]. The Centers for Disease Control and Prevention describe public health surveillance as “the ongoing, systematic collection, analysis, and interpretation of health-related data essential to planning, implementation, and evaluation of public health practice” [2]. Other definitions include the World Health Organization’s conceptualization of surveillance as continued watchfulness and the monitoring of events linked to action [3]. More broadly, Lyon [4] has defined surveillance as “the focused, systematic, and routine attention to personal details for purposes of influence, management, protection or direction.”

One of the outcomes of the COVID-19 pandemic and the corresponding global response has been the rapid development and deployment of digital technologies used for public health surveillance. New and existing digital technologies, including mobile phone location tracking, mobile phone apps, drones, and closed-circuit cameras, have been used for surveillance purposes in supporting the detection and mitigation of disease spread and to enhance compliance with public health measures [5]. The accelerated use of these technologies during the COVID-19 pandemic has led to some scholars describing the COVID-19 pandemic as the first public health crisis of the “digital age” [6] and as a catalyst for a “digital revolution” in surveillance [7].

Despite the extensive use of a plethora of digital solutions for public health surveillance, their actual efficacy in predicting disease spread or supporting public health responses has been unclear [8,9], and there remain ongoing questions about the ethical dimensions of digital surveillance. For instance, the use of digital tools by the state, its agents, the private sector, and other actors has raised concerns around the potential consequences of surveillance, particularly for those who are most marginalized [10-12]. The rapid pace of technological development has also limited opportunities to consider the consequences of technology use or misuse and whether digital technologies are actually effective in mitigating the health-related, economic, and social effects of the pandemic [13].

The growing attention paid to digital surveillance during the COVID-19 pandemic has also offered an important opportunity to reflect on the function of surveillance within public health. For instance, the ubiquitous nature of contemporary modes of data collection enabled by digital surveillance technologies has prompted a reconsideration of pervasive health monitoring [14]. The collection of large amounts of data from sources that have not traditionally fallen within the purview of public health surveillance (eg, transaction monitoring, closed-circuit cameras, drones, and location tracking through mobile devices) presents an urgent need to contend with the consequences of surveillance and the potentially invasive and coercive aspects of public health. Likewise, the use of digital surveillance technologies by a range of actors and for a variety of different objectives throughout the COVID-19 pandemic signals an important opportunity to consider the blurred boundaries between public health surveillance and other forms of surveillance (eg, criminal–legal surveillance, commercial use of data) and how these modes of surveillance and their aims overlap.

This scoping review examined peer-reviewed and gray literature on the global use of digital technologies for public health surveillance during the COVID-19 pandemic to (1) describe the nature of digital technologies used for surveillance during the COVID-19 pandemic, (2) describe the witnessed and potential short- and long-term implications of using digital technologies for public health surveillance, (3) synthesize the peer-reviewed and gray literature regarding the use of such technologies for surveillance during the COVID-19 pandemic response, and (4) identify gaps within this knowledge base. To meet these objectives, the review was guided by two research questions: (1) What is known about digital technologies used for surveillance during the COVID-19 pandemic? and (2) What are the short- and long-term predicted and witnessed impacts of digital surveillance during the COVID-19 pandemic?

## Methods

### Study Design

The protocol used to conduct this research followed the 5-stage methodology described by Arksey and O’Malley [15] and Levac et al [16], which included (1) identifying the research question; (2) identifying relevant studies; (3) study selection; (4) charting the data; and (5) collating, summarizing, and reporting the findings. A summary of the study’s methodology is included below; for a complete description, refer to a previously published scoping review protocol [17]. The study’s reporting structure also conformed to the PRISMA-ScR (Preferred Reporting Items for Systematic Reviews and Meta-Analyses Extension for Scoping Reviews) guidelines and checklist (Multimedia Appendix 1 [18]) [19].

### Data Collection

#### Peer-Reviewed Literature Search

In January and February 2021, the MEDLINE (Ovid), PubMed, Scopus, CINAHL, ACM Digital Library, Google Scholar, and IEEE Xplore databases were searched for peer-reviewed literature. Journals and reference lists of relevant papers identified by our interdisciplinary team of researchers were also reviewed for additional publications. Textbox 1 shows the search terms and strategy created with guidance from a specialist research librarian.

**Search terms developed with the assistance of a health specialist research librarian.**

1. Population Surveillance or Public Health Surveillance, or surveillance.tw.
2. digital surveillance.tw.
3. biosurveillance.tw or Biosurveillance
4. epidemiological monitoring.tw. or Epidemiological monitoring
5. 1 or 2 or 3 or 4
6. pandemic.t.w. or Pandemics
7. disease outbreak.tw. or Disease Outbreaks
8. Coronavirus Infections or covid-19.tw.
9. covid-19.tw.
10. H1N1.t.w.
11. SARS.t.w. or SARS Virus
12. 6 or 7 or 8 or 9 or 10 or 11
13. Public Health or public health application.mp.
14. 5 and 12
15. 13 and 14

An initial broad search was conducted to capture all English-language publications on the use of digital technologies for public health surveillance during previous and current pandemics, epidemics, and outbreaks. Documents were retained if they were published between January 1 and December 31, 2020. This preliminary search yielded 18,449 publications for review. Following the removal of 8819 duplicates, 9630 unique documents were subjected to title and abstract screening by 2 researchers (LC and MND) to assess their fit with the study’s aims. In this initial search, the following inclusion and exclusion criteria were used during the title and abstract screening: (1) document title or abstract mentioned the use of a digital technology for public health surveillance, (2) digital surveillance focused on containing or limiting the spread of an infectious disease, (3) public health surveillance monitored humans rather than nonhuman animals, and (4) digital technology used explicitly for surveillance (eg, data collection).

Consequently, 2076 publications were retained for full review following title and abstract screening. Following a full-text review, 888 publications were retained for analysis.

To conduct a feasible review of the literature, the research team narrowed the scope of the review to focus on COVID-19-pandemic–specific apps of digital technologies for public health surveillance. During this secondary screening, documents were retained for analysis if they were published between December 1, 2019 and December 31, 2020, and if they explicitly included any of the terms such as “coronavirus,” “COVID 19,” “SARS-CoV-2,” or “severe acute respiratory syndrome coronavirus 2” in the title or the abstract. This period was chosen not only to obtain a manageable number of publications for review but also to focus our attention on the first year of the COVID-19 pandemic as a means of better understanding the questions, concerns, findings, and discussions that emerged from the academic and nonacademic literature during this pivotal time. This process led to a final study sample size of 147 unique documents. Throughout this process, in cases in which the 2 screening researchers disagreed or were uncertain whether a publication met the inclusion criteria, a third researcher read the text and discussed with the 2 researchers to decide whether to include the document for analysis.

#### Gray Literature Search

Our search of the gray literature was guided by our multidisciplinary team of researchers as well as a specialist research librarian, who assisted in identifying relevant organizational websites that explore the use of digital technologies for surveillance. These websites included the Ada Lovelace Institute, Human Rights Watch, and the Munk School. The websites of these organizations were searched to retrieve potentially relevant current and archived publications using the same protocol and search terms used to search peer-reviewed literature. Publications were retained for screening if their content related to the use of digital technologies for surveillance during the COVID-19 pandemic. The initial search of the gray literature yielded 141 documents.

Gray literature publications were screened using the same inclusion criteria used to screen peer-reviewed literature. After 2 rounds of screening, 74 documents were retained for analysis. Five conference proceedings identified while searching the peer-reviewed literature were also included as gray literature for a total of 79 publications.

**Figure 1:**
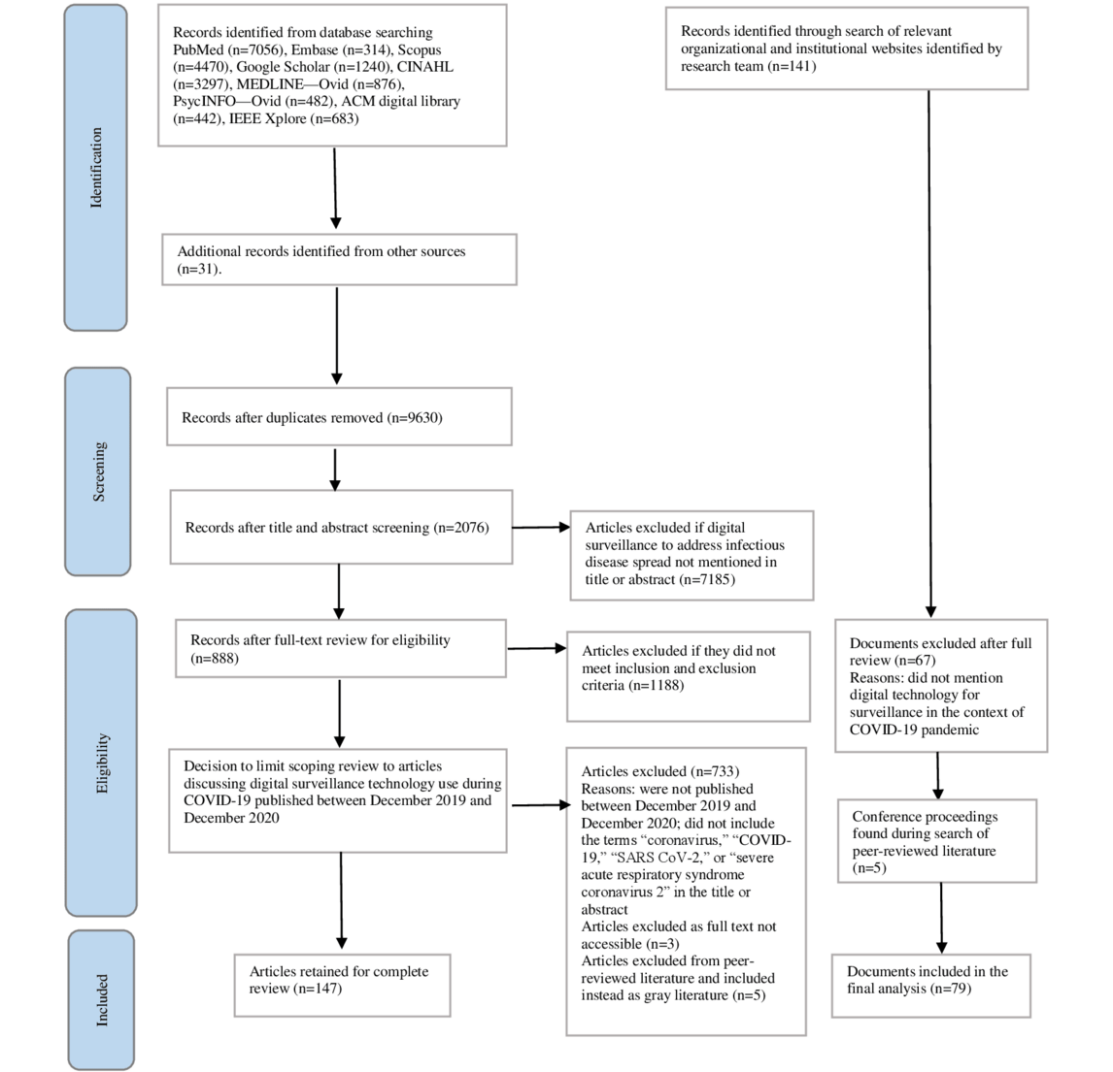
Study selection process.

Due to the breadth and depth of the literature relevant to this review, attention to the social and ethical implications of digital health surveillance for public health purposes during the COVID-19 pandemic response is the focus of this scoping review. A second paper describes the types of digital technologies used during the COVID-19 pandemic response and the factors impacting their effectiveness in public health surveillance [8]. Figure 1 shows the PRISMA (Preferred Reporting Items for Systematic Reviews and Meta-Analyses) chart describing the study’s inclusion process.

### Data Analysis

Documents were analyzed through an inductive coding process to identify dominant themes and patterns [20]. Following an initial analysis of 10 documents and discussion among 5 researchers, an extraction table was developed and further refined upon analysis of 5 additional publications. Five researchers piloted the data extraction table to ensure consistency of analysis and collaboratively reviewed all retained peer-reviewed and gray literature documents for data extraction and analysis. The completed table was used by 5 reviewers to extract data from each publication related to (1) authors; (2) author locations based on affiliated institutions as identified in publications; (3) study aim or objective; (4) methodology and research design; (5) countries or regions identified; (6) types of digital technologies identified; (7) intended or stated outcomes of digital technology use; (8) target populations; (9) unintended (or not explicitly stated) outcomes and implications of technology use; and (10) theoretical analyses, arguments, discussions, and debates. The findings were reviewed and discussed among our multidisciplinary team of researchers to ensure a range of expert perspectives from nursing, medicine, public health, epidemiology, surveillance, geography, health information science, law, bioethics, policy, knowledge translation sciences, critical theory, and sociology of health.

## Results

### Overview

The peer-reviewed literature included commentaries (n=39), reviews (n=39), analyses (n=27), reports (n=8), empirical studies (n=24), legal reviews (n=3), and a policy analysis paper (n=1). The gray literature included commentaries and opinion pieces (n=33); video and audio recordings and transcripts of conferences, panels, and meetings (n=8); news reports (n=33); and conference proceedings (n=5). Identifying the institutional affiliations of authors of peer-reviewed publications allowed us to grasp the international breadth of the literature (authors of more than 1 publication were counted only once). Authors were affiliated with institutions from countries including the United States (n=156), the United Kingdom (n=91), India (n=40), China (n=28), Australia (n=28), Singapore (n=27), Spain (n=25), Italy (n=24), Germany (n=21), Brazil (n=20), Japan (n=19), the Democratic Republic of the Congo (n=16), Switzerland (n=16), Taiwan (n=15), Canada (n=14), Ireland (n=12), South Africa (n=11), and Iran (n=9).

Across the publications reviewed, the use of digital technologies for public health surveillance during the COVID-19 pandemic was identified in 90 countries and regions (Multimedia Appendix 2). Mobile phone apps (particularly apps to support contact tracing), mobile phone location tracking through Bluetooth and GPS technologies, drones, temperature-scanning technologies, and wearable devices were among those most commonly discussed. Digital technologies were most frequently used to support contact tracing (ie, to identify individuals with the disease or symptoms and those with whom they have been in contact) [10,11,21-173]; to inform decision making by public health agencies, states, and individuals [21,25,28,31,44,47,49,51-53,56-60,62,63,66,67,77,81-83,88-90, 94,97,98,102,104,121,129,157-159,167,170,171,174-204]; to monitor compliance with public health measures [21,23,25,28,30,34-38,41,42,44,45,54-58,60,61,63,71,83,85,86,89,91, 94,95,100-102,105,108,110,119,125-127,129,130,135,140,155,156,165,170, 174,182,185,188,190,197,198,205-215]; and to inform the provision of health care services [28,41,44,46,54,57,62,64, 66,69,77,80-82,87,94,95,102,108,126,128,159,170,174, 177,182,183,187,191-193,197,198,201,202,216-224].

### Thematic Analysis

Key considerations related to the use of digital technologies for COVID-19 public health surveillance were grouped into five themes: (1) data security and privacy, (2) function and mission creep related to digital surveillance, (3) consequences of private sector involvement in surveillance, (4) human rights and civil liberties and impacts on marginalized groups, and (5) ethical principles for digital technology design and use.

#### Data Security and Privacy

Many authors identified risks to data security and data privacy associated with the use of digital technologies for public health surveillance (n=138) [11,22-26,28,30,31,33-45,47,48,51, 53,55,57-68,70-77,79-81,83,84,86-92,94-98,101-110,114,116, 118,121-123,125,128-130,132,134,137,147-152,154-165,168,169,172-174,179, 185,187,191,192,195-198,201,203,204,207,208,210-212,216,221,225-232]. Several authors argued that digital surveillance may exacerbate risks to information privacy given that data gathered through digital technologies are increasingly granular and that health data privacy protections often do not adequately address the collection and use of data through digital technologies, such as mobile apps [22,25,37,42,98,110,132,156,162,164,179,228,229]. Others noted that most technologies used for surveillance are products of private companies, and they advised caution related to potential function creep and exploitation (eg, data monetization) of data intended for public health purposes. The idea of a capitalist data strategy is a relatively new aspect of disease surveillance for many public health systems [34,211], and several authors acknowledged that in many countries, including Canada and the United States, health data regulations generally apply to data collection by the state and its agents but do not necessarily extend to private companies [24,33,132,190,195,211].

Issues with data security were also found to present potential risks to data privacy, particularly with regard to technologies that were rapidly deployed without full consideration of potential security vulnerabilities [10,28,33,97,102,105,110,147,150,233]. The UK government, for instance, posted the code for its mobile contact tracing app for public review and received feedback that data logs were not adequately encrypted and risked revealing individuals’ personal information [150]. Likewise, in a review of 50 mobile apps, the authors of another study found that while 30 of the apps required access to features including contacts, photographs, media files, location data, the camera, and call information, only 16 of the reviewed apps indicated that users’ data would be made anonymous, encrypted, or secured [28].

A number of digital technologies used globally during the COVID-19 pandemic were identified as examples of privacy- and security-compromising surveillance. A frequently cited example was the use of cell phone location tracking to support contact tracing and quarantine enforcement in countries including Israel, India, the Czech Republic, China, South Korea, Argentina, Bahrain, Kuwait, and Norway [30,36,42,54,61,89,115,155,190,204,225]. The use of thermal cameras to detect fever among potentially symptomatic individuals in countries including the United States, China, South Korea, Thailand, Singapore, Italy, and the United Kingdom was also identified as a form of surveillance that may reveal sensitive information about underlying health conditions (eg, pregnancy) or stigmatized behaviors (eg, substance use) [44,95,221]. While van Natta et al [221] found that thermal cameras are typically construed as minimally invasive, they cautioned that data regarding individuals’ health could be used by data brokers, advertisers, employers, and law enforcement agencies.

Several authors conceptualized individuals’ right to privacy as a trade-off between data privacy and security and public health. The decision by different states to use centralized or decentralized contact tracing databases, for instance, was frequently mentioned as an example of the tensions between mitigating disease spread, protecting public health, and ending public health measures, such as lockdowns, while also protecting individuals’ data privacy and security [31,34,35,46,112-114,120,121,123,137,139,152,163,211]. While data held in centralized databases can be more readily accessed and analyzed (eg, by public health agencies), they are also more vulnerable to data hacks, data leaks, and other security compromises. In contrast, Christou et al [35] provided an alternative perspective in suggesting that the trade-off between the right to health and the protection of fundamental rights, such as privacy, is a false dilemma. They argued instead that these objectives are intertwined and that human rights standards will strengthen global efforts to mitigate the COVID-19 pandemic because greater human rights protections will heighten users’ trust in surveillance tools. The authors challenged the dichotomy between public health and private information and argued that decisions to infringe upon individuals’ data privacy should be informed by wider discussions of social and political values [96].

#### Function and Mission Creep Related to Digital Surveillance

Several authors (n=30) noted that use of digital surveillance may lead to forms of “creep,” such as mission creep and function creep [21,22,26,28,31,34,36,44,54,61,75,83,86,92,93,95,96,101, 102,123,160,185,190,195,196,210,211,221,226,230]. Broadly, the concept of “function creep” has been used to refer to instances in which there is a slow, nearly imperceptible transformation in a data processing system’s proper or intended activity. Meanwhile, the term “mission creep” has been taken up to identify the expansion of a project or intervention beyond its original aim, particularly if an intervention continues to be used beyond the end of the crisis during which it was implemented. In the literature examined in this paper, discussions of “creep” tended to be largely speculative in nature, as authors pointed to historical instances of emergency surveillance that outlasted the initial crisis or led to the repurposing of surveillance measures (eg, surveillance measures deployed by the United States and other states following the 9/11 crisis) and considered the potential for similar forms of creep that may occur following the COVID-19 pandemic [26,44,55,74,190,226].

While some authors focused on the potential for some states to take advantage of the COVID-19 pandemic to entrench forms of surveillance that might otherwise be seen as extraordinary [28,31,36,115,135,196,210,226], others noted that digital surveillance justified by the need to mitigate the COVID-19 pandemic may normalize surveillance. In this way, civil liberties (eg, the right to privacy and the right to freedom of movement) ceded during the crisis may be difficult to reclaim. Likewise, interventions not normally accepted may be normalized over time [10,11,23,31,36,44,48,76,86,91,93,103,115,135,190, 203,205,209,211]. For instance, Leclercq-Vandelannoitte and Aroles [86] drew on Deleuze’s concept of control societies to analyze the normalization of digital surveillance during the COVID-19 pandemic. The authors considered the ways in which digital technologies (eg, mobile apps to support contact tracing used in China, Australia, Denmark, and Singapore) enable “control societies” as they facilitate insidious tracking of individuals’ everyday activities, which may normalize ubiquitous surveillance.

In some cases, authors identified specific technologies and modes of surveillance that may outlast the COVID-19 pandemic and that may repurpose COVID-19 health-related data. For instance, Kitchin [44,190] referenced the Indian state-sponsored mobile contact tracing app, which may be repurposed for political purposes to monitor and discriminate against certain (unspecified) populations. French et al [45] identified historical examples of racial biases in algorithms and questioned whether COVID-19 health data gathered through mobile contact tracing apps might be used, in the future, by law enforcement against racialized and other vulnerable communities. McDonald [135] described vaccination efforts in Bangladesh that may lead to the establishment of a state-supported digital identity program, including a data directory of vaccinated individuals. Pakes [230] discussed “panic buying” of digital monitoring systems for at-home workers (eg, keyboard-tracking technologies, video-monitoring tools) by unspecified employers and argued that the use of these technologies may normalize surveillance that extends beyond the COVID-19 public health mandate and persists after the pandemic has ended.

Tracking disease and its spread is a core mandate of a well-functioning public health system and critical to mitigating the COVID-19 pandemic. The rapid and unprecedented number of technologies employed to contribute to disease tracking sets the COVID-19 pandemic response apart from all other infectious disease and pandemic responses [211]. As such, many authors (n=46) expressed concern regarding the lack of guidelines related to the collection and use of individuals’ information within contemporary digital public health surveillance practices. They also emphasized the tensions created by the omnipresence of digital public health surveillance technologies and the urgency of their employment, which prohibited full discussion of the impacts and implications of digital surveillance strategies on public understanding and acceptance of surveillance practices [22,23,26,28,29,33-35,43,47,53-55,58,63,75,83,86, 90,92,97,103,109,118,122,123,129,134,142,149,155,160,162,174,188,190, 192,196,209,210,221,225,226,229,232,234].

Concerns were also raised around the existence of national and international laws that justify forms of surveillance and contraventions of human rights during times of emergency that would not be acceptable otherwise. The European Union’s general data protection regulation was cited as an example of a data privacy and security law that allows for nonconsensual surveillance and restrictions on privacy to meet public health objectives during crises [35,55,75,98,196,234]. Several authors warned that states may take advantage of public health emergencies to introduce or expand nonconsensual forms of surveillance, as witnessed in states including India, Israel, South Korea, and Singapore, which framed COVID-19 as a threat that required extraordinary measures, including location tracking through mobile devices [30,31,35,42,55,75,98,210,234].

#### Consequences of Private Sector Involvement in Surveillance

Several authors (n=27) highlighted private sector involvement in digital surveillance for public health and how private interests may lead to invasive, inequitable, or harmful surveillance if data are collected and used for commercial purposes [21,23,24,29,31,35,44,45,58,60,75,86,93,114,115,122-125,162,173,185,190, 195,204,211,213,230]. Many of the digital technologies identified within the literature were developed and deployed through private companies or private–public partnerships. These include mobile contact tracing apps developed by the private sector in partnership with governments and public institutions, such as the Apple–Google App Programming Interface designed to support mobile contact tracing [23,24,33-36,44,45,53,57-59,61,64,66,67,70,72,74, 75,78,83,86,90,94-96,105-107,112,114,144,152,160,190,195,210,229], and the use of aggregated and anonymized data collected by private companies, including telecommunications providers [37,44,55,76,121,125,128,129,154,179,190,210].

Private sector involvement in data collection and use has led to the development of proprietary technologies that are not interoperable and that may contribute to a fragmented public health system [35,44,58,60,66,190]. Other potential harms are associated more broadly with surveillance capitalism, the widespread collection and commodification of personal data by corporations [235], and disaster capitalism, the exploitation of situational crises to establish practices and policies at a time when individuals are distracted (emotionally and physically) or otherwise inattentive and less likely to consider the implications of or offer resistance to newly proposed changes in practice or policy [236]. There is a risk, for instance, that private companies might wrongly profit from the ability to predict when outbreaks will occur (eg, artificially increasing the price of medication) [31,44,45,55,67,75,134,174,190]. Furthermore, the potential and witnessed consequences of digital surveillance identified in the literature were frequently associated with the rapid innovation of digital technologies and rushed implementation of these technologies. Newlands et al [75] argued that rushed innovation is often a feature of disaster capitalism, as private interests seek to profit from crises; in the case of the COVID-19 pandemic, they noted that rushed innovation may heighten the risk of emergency legislation passed to make exceptions for use of these tools without full consideration of the ethical or legal implications [31,33,35,44,75,79,86,113,116,164,207].

#### Human Rights and Civil Liberties and Impacts on Marginalized Groups

The impacts of digital surveillance on human rights and civil liberties were noted by many authors (n=58) [10,23,24,28-30,34-36,38,43,44,53-55,61,67,71,78,89,91,98,104-106, 109,111,115,125-131,135,137,154,157, 173,188,190-192,196,198,204,209,210,212, 215,220,223,226,230,232,233,237]. The erosion of freedom of movement [29,35,41,98,134], freedom of expression [75,130], freedom of association [41,130], and freedom of the press [33] was identified as a potential implication of digital surveillance [36,43,203,204,220]. A number of digital technologies used globally for public health surveillance were identified as impinging on rights and liberties, including digital proximity tracking technologies used in South Korea, Singapore, India, China, Hong Kong, and elsewhere [24,29,61,129,130]; use of wearable technologies (eg, ankle bracelets and trackable bracelets) in the United States, Hong Kong, Bahrain, and India [155,198]; and new advances in digital technology, including biosensors, artificial intelligence, and the Internet of Things [220]. Through the expansion of existing surveillance capacities, Russia and China, for instance, were described as using surveillance technologies, including mobile contact tracing apps, location data from telecommunications companies, and mobile apps for quarantine enforcement, in ways that did not contribute to proposed COVID-19 public health goals and impeded on human rights [125].

In addition to the impacts on human rights and civil liberties discussed above, the implications of digital surveillance for marginalized groups were discussed in many (n=46) publications [10,11,23,31,35,43,45,57,58,62,74,83,86,89,90,93,95,100,111,116,119, 124-126,129,130,133,134,137,145,146, 154,173,183,188,197,205,207,208,210,213,223,226,231,237,238]. Hendl et al [90] summarized this discussion through the recognition that the COVID-19 pandemic has not impacted all people in the same way, and that in addition to the need for epidemiological data on inequities generated by the pandemic response, there is a need to consider the ways in which digital surveillance could exacerbate existing vulnerabilities. A frequently cited example pertained to the use of mobile-based emergency alerts in South Korea sent out by public health authorities. These messages included intimate details regarding people infected with COVID-19 and allowed for easy identification of individuals, many of whom faced stigmatization and discrimination, including members of lesbian, gay, bisexual, transgender, intersex, queer/questioning, and asexual communities [25,41,45,58,145,210].

Other communities were described as rightfully wary of digital surveillance and data collection. Immigrant communities and racialized non-White communities, for example, have experienced long histories of discrimination and oppression through surveillance and may fear an exacerbation of marginalization through COVID-19 public health surveillance [34,58,90]. Likewise, the potential for biases and discrimination embedded into algorithms and technologies may heighten algorithmic oppression such that algorithms expose minorities and vulnerable groups to greater surveillance and arbitrary interventions in the name of public health, as in the case of the South Korean alerts described above [35,44,45,54,59,133,190]. Other highlighted risks included the concentration of surveillance on Black and Latino communities in the United States [52] and the use of data on race, ethnic group, gender, political affiliation, and socioeconomic status to stratify populations and engage in social sorting [44,83,190,226].

#### Ethical Principles for Digital Technology Design and Use

Authors of peer-reviewed and gray literature publications proposed several recommendations for designing and using digital technologies for public health surveillance in ways that minimize harmful consequences.

##### Fit for Purpose and Efficacy

Technologies should produce expected outcomes that will lead to actual benefits for public health and fulfill the needs of public health authorities. There should be evidence of this efficacy, and the potential advantages of digital technologies should be compared with other, less invasive options or nontechnological solutions [10,34-42,57,116-119,122,130,132,133,137,148,190, 223,231,238,239].

##### Proportionality

The potential harms associated with digital surveillance should be proportional to the predicted or witnessed threats. Proportionality should include consideration of the disproportionate impacts of surveillance on marginalized groups [21,23,24,29,35,53,90,92,93,125,126,129,149,154,162,173,196,223,226,229,237].

##### Transparency

All aspects of digital surveillance should be performed such that they are easily observable. Information about how data are collected, used, and shared should be not only readily accessible but also easy to understand [29,31,35,39,43,44,53,63,73,95,97, 102,104,113,117,118,120,125,132,149,154,161,173,178,190,192,196,203, 231,232,240].

##### Temporary

There should be deadlines for data collection, use, and retention, and these should be communicated and upheld. In the case of emergency measures, there should be clearly defined indicators of when the emergency will be considered resolved and when emergency measures will end [21,29,35,36,41,43,53,70,73,75, 97,105,122,126,129,149,154,161,173,179,196,226,229,232,237].

##### Rethinking Consent

There is a need to rethink what “consent” means in the contemporary digital surveillance context. In particular, there is an urgent need for discussions of how to avoid illusions of consent—for example, mandating surveillance to access certain spaces or use of data collected in covert ways for health-related surveillance [22,53,55,75,83,89,97,110,118,123,129,149,162, 188,196,210,226,232,234].

##### Purpose Limitation

Collection and use of data should be limited to public health purposes for mitigating infectious disease spread. Data should not be repurposed [21,22,28,29,31,34,35,41,44,104,110,115, 123,125,126,128,149,159-162,173,190,195,196,210,226,230,237].

##### Privacy and Security

Privacy and data security should be protected through privacy by design and technical tools as well as legislation regulating data collection and use [29,31,34-36,42,53,70,75,83,90, 92,96,98,104-107,110,174,190,192,195,196,204,228,229,234,241,242].

##### Accountability

It should be clear who is responsible for data collection and use. Those engaging in surveillance should be answerable to the public, and individuals should be able to seek redress for harms [29,31,35,44,53,68,83,124,149,160,190,203,230,232,237,240].

##### Civil Society and Public Engagement

Digital surveillance for public health should require ongoing participation by the public and input from those who will be under surveillance. All aspects of surveillance should be discussed and debated through a democratic process [29,31,53,111,116,118,132,205,220,230,239]. Several authors (n=30) raised concerns that the use of digital technologies for public health surveillance may have long-term consequences for governance, the state, and civil society [11,23,25,30,33, 41,43-45,52,55,68,71,79,86,91,95,100,114,123,133,190,203-205, 209-211,220,230].

## Discussion

### Principal Findings

In our review of the academic and gray literature published during the first year of the COVID-19 pandemic, we aimed to explore the use of digital technologies for surveillance; the predicted and witnessed short- and long-term consequences of digital surveillance; and the questions, concerns, findings, and discussions that emerged in this first pivotal year of the pandemic. We found that digital technology use was identified in more than 90 countries and regions for pandemic-related applications, including contact tracing, symptom monitoring, disease tracking, enforcing compliance with public health measures, and supporting the direct provision of health care. We also found a number of consequences related to digital surveillance identified in the literature, including implications for data security and privacy, the potential for function and mission creep, consequences of private sector involvement in surveillance, implications for human rights and civil liberties, and impacts on marginalized groups. Finally, we also discovered ethical principles for digital technology design and use proposed throughout the literature.

Concerns regarding the potential consequences of digital surveillance often intersected. For instance, risks to privacy associated with digital surveillance were discussed by framing privacy as a human right, which, if compromised, may also lead to the undermining of other rights, such as freedom of movement, expression, and association. Likewise, the potential for mission and function creep and the expansion of surveillance were linked to concerns around governance during the COVID-19 pandemic and whether this state of exception will extend beyond the crisis. Notably, discussions regarding mission and function creep drew parallels between other historical instances in which surveillance both extended beyond the initial crisis and captured data for uses other than those originally intended, such as the 9/11 crisis. These discussions highlight the fact that there is significant overlap between public health surveillance and other forms of surveillance (eg, criminal–legal and commercial) in both their objectives and their mechanisms. The blurred line between public health surveillance and other forms of surveillance is rendered particularly visible by digital surveillance, as the use of digital technologies for data collection and analysis highlights the ubiquitous, ongoing, and increasingly normalized nature of surveillance, which is likely to extend far beyond the COVID-19 pandemic.

The associations between public health and other institutional relations and modes of surveillance are captured in the authors’ descriptions of the impacts of surveillance on individuals’ human rights and civil liberties. In South Korea, the use of mobile-based emergency alerts sent out by public health authorities led to stigmatization and discrimination against lesbian, gay, bisexual, transgender, intersex, queer/questioning, and asexual communities; while these alerts were deployed in support of the public health response to the COVID-19 pandemic, the objectives of reducing disease spread and protecting public health do not detract from the invasive and coercive nature of these alert messages, nor their impacts on marginalized groups. Likewise, many authors raised concerns about the capitalist exploitation of public health crises and the repurposing of data to serve private interests. These findings mark the importance of attending to the intersections between public health, public health surveillance, and other forms of social control. These discussions are situated within long-standing debates around freedom of choice and other human rights, the coercive character of public health, and ethical quandaries brought to light by digital surveillance, as explored in this paper.

Recent publications have underscored the urgency of attending to questions of human rights related to digital surveillance during the COVID-19 pandemic, particularly in the case of marginalized groups. Abdelrahman [243], for instance, describes the impacts of digital surveillance during the COVID-19 pandemic on vulnerable groups, including African minority populations in China who had surveillance cameras installed outside their homes by the state. Marshall explores the potential implications of increased digital surveillance during the pandemic for queer sex workers, as enhanced surveillance through closed-circuit television cameras and facial recognition reduced their ability to work anonymously and increased their risk of being criminalized [244]. Our scoping review contributes to these ongoing discussions through a thematic, qualitative analysis that identifies the “slippage” between public health surveillance and other forms of surveillance, including their impacts on marginalized groups, regardless of the stated objectives of surveillance.

One of the most interesting findings to emerge from our review is the emphasis on the differences between digital surveillance and other forms of public health surveillance. While surveillance (including global health surveillance) has long been a critical function of public health [245], the emergence of the COVID-19 pandemic as the first global pandemic of the “digital age” has meant that the uncertainty of navigating this public health crisis was exacerbated by the unprecedented development and use of digital technologies for surveillance [6]. Given the unique context in which the COVID-19 pandemic spread and in which digital technologies were used for public health surveillance, some authors drew on theoretical concepts (eg, Deleuze’s theory of the control society and Foucault’s concept of the panopticon) to analyze the normalization of digital surveillance during the pandemic as these technologies became increasingly ubiquitous [79]. The consequences of emerging differences between digital surveillance and traditional forms of public health surveillance (eg, the huge amount of data collected, the ubiquity and pervasiveness of surveillance, and evolving techniques of data analysis, including machine learning) remain to be fully explored [6,246]. Further research is needed to elucidate the impacts of the normalization and omnipresence of digital surveillance and the implications raised in this review, including data privacy and security, creep, private sector involvement, and human rights.

In light of the ramifications associated with digital public health surveillance, the value of these forms of surveillance must be carefully weighed. Recent publications have suggested that the wide range of digital technologies implemented during the COVID-19 pandemic for surveillance purposes faced a number of barriers preventing their successful implementation, and while many of these digital innovations have yet to be formally evaluated or assessed, there is significant uncertainty around their value [8,9]. It is difficult to determine the impact of digital surveillance on disease transmission, particularly given the challenges around technology uptake, implementation, and consistency. Likewise, it is equally difficult to draw comparisons between countries given the various technologies used, the public health and health care infrastructures in which they were deployed, and the varying objectives toward which these technologies were used, including commercial, criminal–legal, and state objectives. In the face of this uncertainty, it will be important to continue to carefully assess the value of digital surveillance and its intended and unintended consequences. Also critical will be a holistic approach to surveillance that accounts for the blurred boundaries between public health, public health surveillance, and other forms of surveillance and social control.

Our scoping review provides a snapshot of peer-reviewed and gray literature publications from the first year of the COVID-19 pandemic. The scope of the publications included in this review is global, and we included authors affiliated with institutions across diverse regions, signaling preoccupation with these topics worldwide. The large number of publications retained also points to the urgency of these concerns and interest in digital public health surveillance in both academic and nonacademic spaces. Ongoing discussions continue to explore and generate appropriate practices, policies, and legislation to address the challenges associated with digital surveillance [6,246]. The findings of this scoping review contribute to our knowledge of digital technologies used for public health surveillance during the COVID-19 pandemic, the potential and witnessed implications of digital surveillance, and ethical principles for technology design and use. This information is critical for leveraging digital public health surveillance in ways that are ethical and that use data to improve health while minimizing potential marginalization associated with surveillance.

### Limitations

While our review provides important insights into the use of digital technologies for public health surveillance, this study has some limitations. Most significant is the restriction of our focus to the first year of the COVID-19 pandemic, which excludes the use of digital technologies for surveillance after this first year of the pandemic and during other public health crises, including other infectious disease outbreaks. Limiting the review to English-language documents may have also limited our analysis of global digital surveillance. As the COVID-19 pandemic continues and other outbreaks, such as mpox, emerge, this publication represents an early appraisal of existing knowledge. However, the occurrence of new infectious disease outbreaks and the use of new modes of digital surveillance underscore the urgency of reviewing this literature.

### Conclusions

This scoping review explored the potential and witnessed short- and long-term consequences associated with the use of digital technologies for public health surveillance during the COVID-19 pandemic identified in the peer-reviewed and gray literature published during the first year of the pandemic. The review found evidence of concerns raised around risks to human rights and civil liberties, the potential normalization of surveillance, the expansion of the state of emergency of the COVID-19 pandemic, and data security and privacy. The literature also included recommendations for more ethical digital surveillance, including transparency, limited retention and use of data, proportionality, and ongoing efforts to introduce adequate legislation to regulate surveillance.

Although this scoping review focuses on the COVID-19 pandemic, the findings have implications for digital surveillance during other infectious disease outbreaks. The rapid pace of digital technology use and development requires consideration of the impacts of rushed innovation and how legislative, public health, technological, and other mechanisms can be used in striving toward more ethical data collection and use. The consequences and potential harms associated with digital surveillance identified in this review raise important questions about the use of digital technologies for public health surveillance and how these technologies can support public health without impinging on rights, liberties, or democratic processes.

## Appendix

Multimedia Appendix 1 PRISMA-ScR (Preferred Reporting Items for Systematic reviews and Meta-Analyses Extension for Scoping Reviews) checklist.

Multimedia Appendix 2 Table indicating, as identified by publications included in this review, digital technologies used for surveillance by country or region.

## Abbreviations

PRISMA: Preferred Reporting Items for Systematic Reviews and Meta-Analyses
PRISMA-P: Preferred Reporting Items for Systematic Review and Meta-Analysis Protocols
PRISMA-ScR: Preferred Reporting Items for Systematic Reviews and Meta-Analyses Extension for Scoping Reviews

## Data Availability

The data sets generated and analyzed during this study are available from the corresponding author upon reasonable request.
