# Supplementary material for: Short- and Long-Term Predicted and Witnessed Consequences of Digital Surveillance During the COVID-19 Pandemic: Scoping Review
Source: JMIR Public Health Surveill. 2024 May 24;10:e47154. doi: 10.2196/47154 (PMC11129783; doi:10.2196/47154)
Supplement: Multimedia Appendix 2 [file publichealth_v10i1e47154_app2.pdf]

**Country or Region**	**Technologies Being Used for Digital Health Surveillance During Covid-19 Pandemic**
Afghanistan	Mobile phone app
Algeria	Mobile phone app
Argentina	Mobile phone location tracking; mobile phone app
Armenia	Mobile phone app; call records and mobile data
Australia	Mobile phone app; drones; wearable devices; anonymized aggregated local data from telecommunications companies
Austria	Mobile phone app; anonymous aggregated mobile phone GPS data; mobile phone location tracking, mobile phone app based on Apple-Google API; call records
Azerbaijan	Mobile phone app
Bahrain	Mobile phone app; CCTV cameras; Bluetooth-enabled wearable device
Bangladesh	Mobile phone app
Belgium	Mobile phone app; mobile phone location tracking; drones; data from mobile operators
Bolivia	Mobile phone app
Brazil	Mobile phone app; mobile phone phone location tracking; participatory surveillance platform; mobile location data from consumer data
Brunei	Mobile phone app
Bulgaria	Mobile phone app; mobile phone location tracking
Canada	Mobile phone app; crowdsourcing technologies; machine learning; anonymized bulk location data from telecommunications companies; drones; Blockchain app
Canary Islands	Mobile phone app
Chile	Mobile phone app
China	Mobile phone app; mobile phone location tracking; CCTV cameras; drones; online search and social media data; facial recognition technologies; digital trave; cards; big data; QR codes; AI chatbots; temperature scanning technology; crowdsourcing through AI; data from telecommunications companies; infrared cameras; smart doorbells and other smart city technology; medical informatic technology; bar codes on mobile phone; smart sensors at airports, seaports, other infrastructure; patrol robots; wearable devices; financial transaction data
Colombia	Mobile phone app

Croatia	Mobile phone app based on Apple-Google API
Cyprus	Mobile phone app
Czech Republic	Mobile phone app
Denmark	Mobile phone app
Ecuador	Mobile phone app; mobile phone location tracking; SOS Covid tool (data from emergency services, Ministry of Telecommunications, Ministry of Health, mobile service providers, mobile app)
Egypt	Mobile phone app
Estonia	Mobile phone app; ad hoc solution developed with mobile network operators; app combined with ankle bracelet
Ethiopia	COVID-19 monitoring platform
Finland	Mobile phone app; aggregated and anonymized data from mobile network company
France	Mobile phone app; CCTV cameras; Google Trends data; data from mobile operators; video surveillance systems equipped with AI algorithms
Georgia	Mobile phone app
Germany	Mobile phone app; aggregated mobile data from telecommunications companies; population mobility data collected from private companies; Google Trends data; call records; app and associated smartwatch; wearable devices
Ghana	Mobile phone app; centralized registry of mobile devices; drones
Gibraltar	Mobile phone app
Guatemala	Mobile phone app; mobile phone location tracking
Hong Kong	Wearable devices; mobile phone app; mobile phone location tracking
Hungary	Mobile phone app
Iceland	Mobile phone app
India	Mobile phone app; mobile phone location tracking; CCTV cameras; recycled anti-terrorism and war surveillance measures; facial recognition software; command and control centers in smart cities; mobile tower signals from ISPs; location and temperature-monitoring bands; cell phone records; geomapping of quarantine locations; geospatial solutions; biometric surveillance systems

Indonesia	Mobile phone app
Iran	Mobile phone app ; mobile phone location tracking; Web-based platform combining informatics and services; Google Trends data
Ireland	Mobile phone app; automated text messaging; app using Apple-Google API; remote monitoring solutions
Israel	Mobile phone app; intelligence tools used for anti-terrorism; geolocation and proximity data; call record data; mobile phone location tracking; credit card purchase data; cyber-monitoring system; thermal cameras; wearable devices
Italy	Mobile phone app; mobile phone location tracking; aggregated movement data from telecommunications companies; crowdsourced data; mobile app based on Apple-Google API; Google Trends data; call record data; thermal cameras mounted on drones; facial recognition technology; data from airline transportation networks; voice recognition software; drones
Japan	Mobile phone app; wearable devices; web search query logs from multiple devices and user location information from location-aware mobile devices; COOPERA chatbot (Covid-19: Operation for Personalized Empowerment to Render Smart Prevention and Care Seeking)
Jordan	Mobile phone app
Kazakhstan	Mobile phone app; mobile phone location tracking
Kenya	Mobile phone app
Kuwait	Mobile phone app
Kyrgyzstan	Mobile phone app
Latvia	Mobile phone app based on Apple-Google API
Liechtenstein	Wearable devices
Lithuania	Mobile phone app
Malaysia	Mobile phone app
Mexico	Mobile phone app; crowdsourcing technologies
Morocco	Mobile phone app; mobile phone location tracking
Nepal	Mobile phone app
Netherlands	Mobile phone app; Google Trends data
New Zealand	Mobile phone app; mobile phone location tracking; online assessments and symptom monitoring tools; QR codes
Nigeria	Electronic forms for contact data collection

North Macedonia	Mobile phone app
Norway	Mobile phone app; telecommunications data
Oman	Mobile phone app
Pakistan	Mobile phone app; mobile phone location tracking
Peru	Mobile phone app; data from call centers and text messaging systems
Philippines	Mobile phone app
Poland	Mobile phone app based on Apple-Google API; mobile phone location tracking; QR codes
Portugal	Mobile phone app
Qatar	Mobile phone app
Romania	Mobile phone app
Russia	Mobile phone app; mobile phone location data; geolocation and proximity data; surveillance cameras equipped with facial recognition tech; QR codes; drones; digital travel permits; facial recognition technologies
Rwanda	Drones
Saudi Arabia	Mobile phone app; electronic health records
Singapore	Mobile phone app; crowdsourcing technologies; drones; location tracking; wearable devices; QR codes; health IT infrastructure; web-based staff surveillance system; contact tracing token; Internet of Things including smart distancing sensors; facial recognition software; AI-equipped temperature screening system; robot dog; geolocation data; text message alert system; SGDormBot to monitor migrant workers; CCTV footage; chatbots; open database of personally identifiable cases
Slovakia	Mobile phone app
South Africa	Mobile phone app; mobile phone location tracking; drones; QR codes; tracking database
South Korea	Mobile phone app; mobile phone location tracking; transaction data; GPS data; satellite data; temperature tracking; surveillance footage, facial recognition technology; health IT infrastructure; emergency text message alert system; visa information and airline and passenger data; overseas roaming data; smart management system; data from telecommunications companies; medical records; wearable devices; cell phone records; fever checks; geofencing; smart city integrated technology; data from mobile phone carriers,

	immigration services, police databases, credit card companies, public transit, government agencies, health insurance companies, and hospitals; AI
Spain	Mobile phone app; mobile phone location tracking; drones; Google Trends data; CCTV cameras; wearable devices; hospital epidemic tracking system; drones with loudspeakers; data from mobile operators; digital health passport developed with PwC
Sweden	Mobile phone app
Switzerland	Mobile phone app; mobile phone location tracking; telecommunications data
Taiwan	Mobile phone app; health insurance database; health IT infrastructure; immigration and customs database; mobile phone location tracking; wearable devices; AI; big data and analytics; data from ID card; infrared temperature checkpoints; GPS functionality and cameras on smartphones; chatbot; GPS in shuttle buses; credit card transactions; CCTV cameras with license plate recognition; highway electronic toll collection system; machine learning; geofencing; digital platforms; integrated IT and big data systems
Thailand	Mobile phone app; data about travelers from high-risk countries; hospital information database; SIM cards; mobile phone location tracking; fever-detecting cameras
Tunisia	Mobile phone app; drones
Turkey	Mobile phone app; mobile phone location tracking
Uganda	Digital contact tracing system
Ukraine	Mobile phone app
United Arab Emirates	Mobile phone app; CCTV cameras; smart helmets used by police
United Kingdom	Mobile phone app; anonymized aggregated local data from telecommunications companies; mobile phone location tracking; drones; crowdsourcing; electronic health records; mobile app based on Apple-Google API; GPS data from private companies including Uber and Facebook; Google mobility reports; syndromic surveillance system including telephone calls, online assessments, health data coding indicators; video cameras with AI capabilities; Google Trends data; patient

	data; private company data analytics and modeling; fever detection cameras; Internet-of-Things
United States of America	Mobile phone app; mobile phone location tracking; crowdsourcing technologies; electronic medical records; social media data; Google Trends data; airline passenger and crew data; movement data from private companies; Apple-Google API; GPS data from private companies including Uber and Facebook; health monitoring platforms; automatic web-based symptom monitoring tool; wearable devices; facial recognition technology; data mining technology; contact tracing call centre with virtual assistants; GPS data from apps on mobile phones; private company data analytics and modeling; CCTV cameras; AI; anonymized bulk location data from telecommunications companies; social distancing detection software; voice recognition; chatbots; data from mobile advertising companies; Google Maps; screening app and website developed with Apple; real-time tracking map
Uruguay	Mobile phone app
Vietnam	Mobile phone app
